# Early changes in macular optical coherence tomography parameters after Ranibizumab intravitreal injection in patients with exsudative age-related macular degeneration

**DOI:** 10.1186/s40942-018-0109-z

**Published:** 2018-02-05

**Authors:** Nicole Antunes de Almeida, Osias Francisco de Souza

**Affiliations:** Centro Médico de Oftalmologia/ProRetina CAPC – University of Campinas School of Medical Sciences, Rua Engenheiro Carlos Stevenson 66, Campinas, SP 13092132 Brazil

**Keywords:** Age-related macular degeneration, Optical coherence tomography, Ranibizumab, Subfoveal exsudative, Choroidal neovascularization

## Abstract

**Background:**

Evaluation of the impact of different macular optical coherence parameters on visual acuity as early as 1 day after injection of ranibizumab in patients with subfoveal exsudative age-related macular degeneration.

**Methods:**

This was an interventional, non randomized, open label prospective study, where we evaluated 20 eyes of 20 patients affected by exudative age-related macular degeneration. These patients were treated with injections of ranibizumab between February 2013 and January 2015. The primary endpoint of this study was to evaluate the early changes in optical coherence tomography parameters (retinal thickness, central and total retinal volume) and impact on best-corrected visual acuity (BCVA) obtained by logarithm of minimum resolution using ETDRS protocol in patients treated with a single dose intravitreal injection of ranibizumab (0.5 mg/0.05 mL) during the first month of follow. The patients were evaluated on the first day, them at 7 and 30 days after the treatment. The National Eye Institute Visual Functioning Questionnaire was applied during the study period to assess early perception of ranibizumab injection effectiveness. The adverse events were monitored throughout the study.

**Results:**

Central retinal thickness values at 1 (464.0 ± 97.8 µm), 7 (379.9 ± 107.8 µm) and 30 days (365.5 ± 95.1 µm) after ranibizumab injection showed a statically significant reduction when compared with baseline results (*P* = 0.01, *P* = 0.001, *P* = 0.001, respectively). Similar alterations were observed in central and total retinal volume, which were detected early on the first day of evaluation, after the measurement at baseline (central: 0.36 ± 0.07 vs. 0.40 ± 0.10 mm^3^, *P* = 0.01; total: 9.62 ± 1.10 vs. 9.99 ± 2.56 mm^3^, *P* = 0.002) and remained steady at 7 (*P* = 0.001, *P* = 0.002, respectively) and 30 days (*P* = 0.001, *P* = 0.004, respectively) with slight variations without losing their gains in these parameters. The best-corrected logarithm of minimum angle of resolution (logMAR) showed a statistically significant difference when compared to the baseline. (0.81 ± 0.16 vs. 0.67 ± 0.24, *P* = 0.005). The NEI-VFQ-25 questionnaire demonstrate statically significant results after treatment. When patients were asked about the subjective improvement in visual quality, over 80% reported early improvement. Throughout the period of follow-up visits, no serious adverse events were reported.

**Conclusion:**

Intravitreal injection of ranibizumab can produce early changes in optical coherence tomography parameters and an improvement in perceived visual quality of patients with subfoveal exsudative age-related macular degeneration.

## Background

Age-related macular degeneration (AMD) is a common cause of central visual loss and legal blindness in older adults. It is most common in individuals over the age of 65 years, occurring in approximately 25% in this age group [1]. The two most common forms of AMD are known as dry (i.e. atrophic) and wet (i.e. subfoveal exsudative). The exudative form is characterized by growth of choroidal neovascularization (CNV), causing hemorrhagic or serous subretinal and/or pigment epithelium detachment and is responsible for over 90% of cases of severe visual loss [1–3].

CNV development involves a series of factors such as inflammation and angiogenesis, leading eventually to sub retinal fibrosis. Vascular endothelial growth factor (VEGF) has been considered an important factor in the development mechanism of CNV. Different treatments targeting VEGF have shown improvement of vision in subfoveal exsudative AMD patients and VEGF inhibition is now considered as an effective strategy for the treatment of this condition [4–6].

Ranibizumab is a high-affinity recombinant monoclonal antibody fragment (Fab) that neutralizes all biologically active isoforms of VEGF-A. It was approved by the FDA (Food and Drug Administration) in June 2006 for treatment of all subtypes of neovascular AMD and it has been proven clinical effective in improving mean visual acuity in several clinical trials [5, 7]. Many diagnostic modalities can be used for detection of CNV in AMD patients. Time domain and spectral-domain optical coherence tomography (SD–OCT) and fluorescein angiography (FA) still remain the best methods to detect CNV [8].

Quantitative OCT analysis, a non-invasive imaging technique, is used to track treatment outcomes in patients with subfoveal exsudative AMD and may play an increasingly important clinical role in the development of anti-VEGF therapies. There are few studies available that have attempted to systematically examine the correlation between OCT-derived morphologic parameters and visual acuity. However, previous studies have reported significant early visual and OCT benefits (within 7 days and a month, respectively) after treatment with an anti-VEGF agent (bevacizumab), even with a low resolution technique. Those studies however used time domain OCT, a technology with lower resolution and poor reproducibility of radial scans, so the information about retinal and subretinal morphology is limited [9–11].

Considering the importance of evaluating the impact of different OCT parameters on visual acuity in the first week after injection of ranibizumab in AMD patients, we used an equipment with SD-OCT capability to obtain images to define which of these parameters correlates better with visual acuity and prognosis [12–14]. Furthermore, since there is no data about patients’ perception of the effectiveness of ranibizumab at an early stage of treatment, we applied the National Eye Institute Visual Functioning Questionnaire (NEI-VFQ-25) during treatment [15].

## Methods

This trial was an interventional, non randomized, open label prospective study with a single arm of treatment. The study and informed consent were approved by the local ethics committee at Medical School of the State University of Campinas. Patients older than 50 years of age, with BCVA between 20/40 and 20/320, determined by ETDRS chart, and primary choroidal neovascularization associated with AMD (type 2) were included. Patients who had other ocular disorders/procedures that could confound interpretation of results, or had reasonable condition that contraindicate the use of this investigational drug, who required ocular surgical procedures during 12 month study period, or needed concomitant therapy with topical ocular or systemically corticosteroids within 4 months prior, were excluded. Previous treatment with anti-angiogenic drugs (pegaptanib sodium, anecortave acetate, bevacizumab, ranibizumab, etc.), known hipersensitivity to ranibizumab and any component of formulation, or inability to comply with study/follow-up procedures, were also considered exclusion criteria.

To establish a diagnosis of active choroidal neovascularization, the presence of leakage would have to be detected by fluorescein angiography test, and the fluid observed either inside or below the retina by SD-OCT. After discussion of the pathogenesis of AMD, the alternatives to therapy, potential risks of treatment with ranibizumab, and prior to receiving the study drug injection, all patients who were considered eligible gave consent for the use of their data for research purposes.

The participants were also asked to complete the NEI-VFQ-25 form for assessment of the visual impairment and symptoms in order to verify the influence of treatment on improving visual impairment and visual symptoms such as the emotional and visual function. This form is a reliable and validated instrument, especially useful in settings such as clinical trials where interview length is a critical consideration. The NEI-VFQ-25 includes 25 core items to measure 12 domains of vision function. In this trial, we used a previously validated Portuguese version of the NEI-VFQ-25, which was applied at the baseline visit, and at 1, 7, and 30 days after administration.

Prior to drug injection, the BVCA for each studied eye was obtained by LogMAR using the ETDRS visual acuity protocol and the presence of leakage from CNV involving the fovea was confirmed by the FA test. Central retinal thickness was assessed twice to 1 mm from the center circle using 7 lines scan protocol and dense protocol using high resolution SD-OCT (Heidelberg Spectralis, version 5.1, Germany). Line scanning images were also used for examine morphological features of retinal including cystoid spaces, retinal thickening, sub retinal fluid or pigment epithelial detachment.

After preparation of the eyelids with a 10% povidone-iodine swab and instillation of 5% povidone-iodine in the lower conjunctival fornix, the eye was anesthetized with topical 1% tetracaine chloridrate. A lid speculum was used for access to the eye with minimal pressure on the globe and 0.5 mg/0.05 mL of ranibzumab (Lucentis^®^, Novartis Pharma AG, Switzerland) was injected intravitreally with a 30-gauge needle followed by administration of gatifloxacin ophthalmic solution. The permeability of the central retinal artery was verified by ophthalmic assessment and the intraocular pressure expected before discharge of a participant was < 30 mmHg. Participants received instructions for the administration of a topical antibiotic for a period of 5 days after this procedure.

In order to evaluate change in study parameters, the data collected included a full ophthalmological exam, BCVA and retinal imaging with quantitative SDOCT parameters (foveal thickness, foveal volume—central 1 mm circle, retinal volume at 3 and 6 mm central circles), and the completion of the NEI-VQF-25.

The software programms used for statistical analysis were SPSS V.17 (DMSS Software^®^—São Paulo—Brasil), Minitab 16 (Minitab Inc. USA) and Microsoft Excel (2010). We considered the significance level of 0.05 (5%) with a confidence interval of 95% (CI 95%) for this study. A non--parametric test (Wilcoxon, Friedman and Equality test) was chosen due to the small sample (less than 30 individuals).

## Results

Twenty eyes of 20 consecutively participants (11 women and 9 men), with a mean age of 76.1 ± 8.7 years and a clinical diagnosis of exudative AMD were selected for this trial. All patients selected were submitted to a Ranibizumab intravitreal injection between February 2013 and January 2015 (Table 1).

**Table 1:** Demographic characteristics

	Characteristics	Result
1	No of eyes	20 (one of each participant)
2	Age (mean ± SD)	76.1 ± 8.7 years; IC (72.3; 79.9)
	Age (median)	75.5 (range 55–93 years)
3	Gender	11 women, 9 men
4	Eye	14 OD, 06 OS
5	Characteristic of crystalline lens	15 IOL, 05 translucent
6	PED	09 no, 11 yes
7	Presence of drusen	3 no, 17 yes

*IOL* intraocular lens, *PED* pigment epithelial detachment, *OD* right eye, *OS* left eye

The changes in BCVA log MAR values from baseline to 1, 7 and 30 days after injection are shown in Table 2. The comparison with the baseline values (0.81 ± 0.16) showed a gradual increase in BCVA observed after ranibizumab intravitreal injection (0.72 ± 0.23 at day 7), becoming even more evident after 30 days (0.67 ± 0.24) with statistically significant difference (*P* = 0.005).

**Table 2:** The changes in BCVA/LogMAR measurements

	Time after injection	Mean ± SD	IC95%	*P* value
1	Baseline (prior injection—T0)	0.81 ± 0.16	0.74; 0.88	–
2	1 day after (T1)	0.76 ± 0.19	0.67; 0.85	0.066^a^
3	7 days after (T7)	0.72 ± 0.23	0.61; 0.83	0.016^b^
				0.157^c^
4	30 days after (T30)	0.67 ± 0.24	0.55; 0.78	0.005^d^
				0.028^e^
				0.109^f^

^a^T0/T1; ^b^ T0/T7; ^c^ T1/T7; ^d^ T0/T30; ^e^ T1/T30; ^f^ T7/T30

A month after ranibizumab injection the values obtained for central retinal thickness showed a significant decrease, as a consequence of reduction of subfoveal fluid (under internal retina). The retinal thickness values started at 524.5 µm and decrease to 353.0 µm on day 30 (*P* = 0.001) (Table 3).

**Table 3:** Changes in central retinal thickness over a month (*n* = 20)

	Time of injection	Mean ± SD (µm)	Median (µm)	*P* value
1	Baseline (prior injection—T0)	512.3 ± 126.9	524.5	–
2	1 day after injection (T1)	464.0 ± 97.8	458.5	0.014^a^
3	7 days after injection (T7)	379.9 ± 107.8	351.0	0.001^b^
				0.001^c^
4	30 days after injection (T30)	365.5 ± 95.1	353.0	0.001^d^
				0.001^e^
				0.179^f^

^a^T0/T1; ^b^ T0/T7; ^c^ T1/T7; ^d^ T0/T30; ^e^ T1/T30; ^f^ T7/T30

Immediate decrease in central retina volume (CRV) (0.41–0.36 mm^3^) and total retinal volume (TRV) (9.94–9.34 mm^3^) were seen early on the first day of evaluation and reduced more at days 7 and 30 with minor variations (Tables 4 and 5).

**Table 4:** Change in central retinal volume over a month by SD-OCT(*n* = 20)

	Time of injection	Mean ± SD (mm^3^)	Median (mm^3^)	*P* value
1	Baseline (prior injection—T0)	0.40 ± 0.10	0.41	–
2	1 day after injection (T1)	0.36 ± 0.07	0.36	0.011^a^
3	7 days after injection (T7)	0.29 ± 0.07	0.28	0.001^b^
				0.003^c^
4	30 days after injection (T30)	0.29 ± 0.08	0.28	0.001^d^
				0.002^e^
				0.265^f^

^a^T0/T1; ^b^ T0/T7; ^c^ T1/T7; ^d^ T0/T30; ^e^ T1/T30; ^f^ T7/T30

**Table 5:** Change in total retinal volume over a month by SD-OCT(*n* = 20)

	Time of injection	Mean ± SD (mm^3^)	Median (mm^3^)	*P* value
1	Baseline (prior injection—T0)	9.99 ± 2.56	9.94	–
2	1 day after injection (T1)	9.62 ± 1.10	9.34	0.002^a^
3	7 days after injection (T7)	8.77 ± 0.83	8.48	0.002^b^
				0.001^c^
4	30 days after injection (T30)	8.75 ± 1.39	8.30	0.004^d^
				0.028^e^
				0.391^f^

^a^T0/T1; ^b^ T0/T7; ^c^ T1/T7; ^d^ T0/T30; ^e^ T1/T30; ^f^ T7/T30

Although of NEI-VFQ-25 questionnaire results did not demonstrate significant change before and after treatment, except when asked about the subjective improvement in visual quality, over 80% of patients reported early improvement.

Intraocular pressure was measured before drug administration and after 7 days (T0, T7 and T30). No clinically significant change from baseline can be observed in IOP values (*P* = 0.062). Throughout the period of follow-up visits, no serious adverse events were reported.

## Discussion

Symmetrical distribution and a small variability (variation coefficient < 50%) in the population was considered a positive point for results validation, as demonstrated in the demographic data.

Overall, treatment with ranibizumab provided clinically and statistically significant improvement in subfoveal exsudative AMD [15–17]. Corroborating others, our results of changes in the SD-OCT parameters and the visual acuity in patients with AMD after the first intravitreal injection of ranibizumab (0.5 mg) demonstrated a statistically significant difference over 30 days, which may be verified by the reduced values of the retina thickness and lowering the total volume of the central retina.

However, we also found statistically significant difference in the days 1 and 7. Pharmacokinetic studies about anti-vegf described relatively long half-lives (7–10 days) after intravitreal depot injections and clinical durations of action that usually exceed 4 weeks [16, 18]. Therefore, it is not the peak level of drug that will dictate efficacy after 1 month, but rather the amount of residual drug present in the eye 1 month after the injection, which is also known as the trough level [17]. It seems reasonable to assume that these eyes may initially respond well.

Despite the importance of these findings, the primary aim of this study was to access the early changes in volume and retinal thickness using SD-OCT and its possible impact on visual acuity. LogMAR BCVA results showed a significant improvement within the first day and 1 month after only one ranibizumab intravitreal injection (*P* < 0.05). In this context it was possible to observe the immediate effect of ranibizumab in reducing thickness and volume of retina, which started early (day 1) and persisted through days 7 and 30 with slight variations.

The persistence of these effects in reducing the thickness and volume of the retina suggests that a clinician could utilise Ranibizumab intravitreal injections on a 30 days interval as a safe protocol for AMD.

Also, in a phase I/II study with neovascular AMD patients, a repeated treatment with intravitreal injections of ranibizumab for up to 3 months were associated with an improvement in visual acuity and decreased leakage from choroidal neovascularization in these population, corroborating the hypothesis that a long-term evaluation with repeated doses of drugs could demonstrate better results [19, 20].

The NEI-VFQ-25 questionnaire results demonstrate significant alteration before and after treatment when patients were asked about the subjective improvement in visual quality. The results also showed that over 80% of patients reported early improvement as a high motivation to continue treatment.

## Conclusion

In the present study we observed that a single intravitreal injection of ranibizumab can produce significant early changes in SD-OCT parameters and improve visual acuity in patients with subfoveal exsudative AMD, contributing to subjective confidence and consequently compliance with a long term treatment and follow up.

## Abbreviations

AMD: age-related macular degeneration
BCVA: best-corrected visual acuity
CNV: choroidal neovascularization
ETDRS: early treatment diabetic retinopathy study
FA: fluorescein angiography
Fab: monoclonal antibody fragment
IOL: intraocular lens
LogMAR: logarithm of minimum angle resolution
NEI-VFQ-25: national eye institute visual functioning questionnaire
PED: pigment epithelial detachment
SD-OCT: spectral domain optical coherence tomography
VEGF: vascular endothelial growth factor
